# Elementary flow mapping across life cycle inventory data systems: A case study for data interoperability under the Global Life Cycle Assessment Data Access (GLAD) initiative

**DOI:** 10.1007/s11367-024-02286-x

**Published:** 2024-03-12

**Authors:** Antonio Valente, Carl Vadenbo, Simone Fazio, Koichi Shobatake, Ashley Edelen, Thomas Sonderegger, Selim Karkour, Oliver Kusche, Edward Diaconu, Wesley W. Ingwersen

**Affiliations:** 1 European Commission, Joint Research Centre, Directorate D–Sustainable Resources, 21027 Ispra (VA), Italy; 2 ecoinvent Association, Technoparkstrasse 1, 8005 Zurich, Switzerland; 3 TCO2 Co. Ltd., 6F Daigonagamori Bldg., 12 Nandocho, Shinjuku-ku, Tokyo 162-0837, Japan; 4 Eastern Research Group (ERG), Cincinnati, OH 45219, USA; 5 Oliver Kusche Research & Consulting, Marie-Curie-Str. 1, 79104 Freiburg, Germany; 6 Center for Environmental Solutions and Emergency Response, US Environmental Protection Agency, Cincinnati, OH, USA

**Keywords:** Nomenclature alignment, Data accessibility, Mapping framework, Data harmonization, Life Cycle Initiative, Flow list alignment

## Abstract

**Purpose:**

Limited availability of life cycle assessment (LCA) data poses a significant challenge to its mainstream adoption, rendering it a central issue within the LCA community. The Global LCA Data Access (GLAD) network aims to increase the accessibility and interoperability of LCA data and offers benefits for different use cases. GLAD is an intergovernmental collaboration involving different stakeholders organized into working groups. The GLAD Nomenclature Working Group (NWG) developed a procedure and a set of criteria to map elementary flows among major nomenclature systems and reviewed bidirectional mappings. This paper provides an overview of the methodological approach followed by the NWG to achieve the resulting mapping files.

**Methods:**

The mapping procedure involves several steps of flow and compartment matches and bilateral review. The procedure is supported by an ad hoc software tool called the “GLAD Mapper Tool” developed with the NWG and which is made available for free by the European Commission. The input files for the procedure are the properly formatted source and target flow lists and a file containing the mapping criteria. The four nomenclature systems mapped are those used in ecoinvent, Environmental Footprint, IDEA, and the U.S. Federal LCA Commons. The procedure included representatives from each of these nomenclature systems to ensure a multilateral agreement on the approach to verifying and assessing the quality of the results. The iterative mapping process included different stages of bidirectional reviews to achieve a balance between mapping coverage (i.e., percentage of source flows covered by the target list) and accuracy.

**Results and discussion:**

The mapping procedure proved to be an efficient approach for LCA practitioners in mappings between different nomenclature systems. After a relatively low number of iterations, mapping coverages higher than 90% were achieved, which is driven by the availability of unique substances (flow names) and the granularity of environmental compartments. Overall, none of the four flow lists achieved full coverage and the use of approximated matches (proxy matches) for environmental compartments and/or substances was necessary when a perfect matches between flows were not possible.

**Conclusions:**

The NWG’s mapping activities may serve as a starting point towards defining a central hub for mapping impact assessment methods and datasets, improving data accessibility and interoperability for the LCA community as a step towards defining a unified nomenclature system. The GLAD mapping approach is open and transparent. The approach fosters traceability in the mapping process and offers the potential for greater interoperability across the LCA community, underlining the commitment to openness and collaboration.

## 1 Introduction

Life cycle assessment (LCA) methodology is widely used in various fields to evaluate the environmental impact of human activities and to support decisions and strategies in areas like energy system decarbonization, circular economy, sustainable production and consumption, and finance (Sala et al. 2021; Hellweg et al. 2023). The need for increased data access became even more pressing due to the expansion of LCA in different situations of application-specific data availability. LCA practitioners must be able to identify and access the most appropriate life cycle inventory (LCI) data for proper modeling and analysis. However, poor data availability and accessibility might hinder the mainstream uptake of LCA (e.g., by users from industry, academics, and governments). Data availability is often not sufficient to guarantee the appropriate representativeness of the system in the scope of the analysis. Furthermore, as highlighted in the critical review by Edelen et al. (2018), LCI data are developed worldwide by multiple sources using different nomenclature systems (i.e., systems of names, terms and rules) for contexts (i.e., environmental compartment information such as “emission to air, indoor” or “resources, from ground”) and flow names (i.e., matter, energy, or space entering or leaving the technosphere or biosphere irrespective of the context). These differences hinder efficient data exchange or integration between different data sources increasing the risk of inconsistencies when it comes to characterizing the environmental performance by matching the characterization factors (CFs) of a life cycle impact assessment (LCIA) method with a LCI of datasets. In this sense, the interoperability of data becomes a critical aspect for both human and machine readability in LCA studies.

The Global LCA Data Access (GLAD) network was launched in 2002 under the Life Cycle Initiative hosted by UNEP (https://www.lifecycleinitiative.org), GLAD’s mission is to enhance the accessibility and interoperability of LCA data. This initiative provides benefits across various applications, including supporting companies in making green claims, disclosing accurate product-related environmental information, and contributing to public policies related to climate change, circular economy, and environmental labeling. The GLAD initiative is an international collaboration involving members from numerous countries and approximately a hundred stakeholders organized into three main working groups (WGs): (i) Network Architecture and Technology WG (NATWG), (ii) Metadata Descriptors WG (MDWG), and (iii) Nomenclature WG (NWG) (Milà i Canals et al. 2016). Under the GLAD umbrella, each Working Group (WG) conducts specific activities and provides insights and conclusions to the GLAD Steering Committee. Thanks to the coordinated efforts of the three interlinked Working Groups (WGs), the GLAD initiative launched the functional platform (https://www.globallcadataaccess.org) in 2020, enabling users to search, filter, compare, and access LCA datasets from different sources in a centralized user interface. Within the GLAD framework, data providers are required to adhere to a set of minimum metadata descriptors and use a common data exchange format to ensure data interoperability.

Within the GLAD context, interoperability of LCA data is a core concern, and one of the goals of the NWG’s work is to establish a proper procedure for translating process datasets from one nomenclature system to another. As noted in the work of Edelen et al. (2018), the quality and reliability of the match (e.g., when matching an LCI with an LCIA) depend on the strategy and business of the two nomenclature owners (which are often independent of each other) potentially leading to a loss of data integrity and consistency. In their critical review, Edelen and colleagues provided a list of recommendations for LCA flow list owners to foster improvements on their lists in terms of clarity, translatability, and robustness to enhance interoperability and the integrity of the LCA data network. In their work, the authors specified that further recommendations are delegated to the GLAD NWG.

The present paper focuses on the activities related to the NWG exclusively. The main scope of the NWG is to overcoming the interoperability problem that exists between LCA data sourced in different reference nomenclatures. As a first step, the NWG defined a set of criteria and applied them to map the elementary flows among the major international nomenclature systems. Notably, typical interoperability issues between nomenclature systems arise when different names are given to the same substance (e.g., under two hypothetical nomenclature systems, fossil carbon dioxide emissions may be named “fossil CO_2_” and “carbon dioxide, fossil”) or when there are differences between the environmental compartments of nomenclature systems (e.g., emission to industrial soil may be modeled as “emissions/ground/industrial” in a reference nomenclature, but as “emissions/soil/non-agricultural” in another). Furthermore, from a software point of view, differences in characters might bring about interpretation confusion (e.g., the strings “N2O” and “N_2_O” may be differently interpreted by LCA software). Considering that the number and types of both flows and environmental compartments (*contexts*) covered in nomenclature systems may vary by orders of magnitude, the achievement of an acceptable level of correspondence (both in terms of percentage of coverage and quality of the matches) between the items of different flow lists is often a challenging and time-expensive task.

In a work led by the EC Joint Research Centre (Sanyé-Mengual et al. 2022), mapping rules between LCIA methods and LCIs were defined and applied to seven LCIA methods addressing biodiversity. The LCIA methods, provided in their native nomenclature system, have been matched with the Environmental Footprint v3.0 and ecoinvent v3.6 flow lists. In Sanyé-Mengual et al. the mapping procedure between LCIA and LCI was defined and applied to contribute to the operationalization of LCIA methods and models (in the use-specific case, LCIA methods address biodiversity impacts). The analysis of the resulting mapped methods and uncharacterized elementary flows showed a significant difference in terms of final coverage between the two nomenclature systems with potential discrepancies on the interpretation of the results (Sanyé-Mengual et al. 2023).

Overall, the works of Sanyé-Mengual et al. and Edelen et al. (both closely linked to the activities of the GLAD NWG) represent an important background to the NWG goals. Notably, both highlight that complementing the base elements of nomenclature systems (flow name names, UUIDs, compartments, and units) with clear and consistent pieces of machine-accessible information (such as CAS numbers, synonyms, and formulas) is pivotal not only for reaching higher matching rates but also for reducing the efforts required (e.g., by allowing automatic-systematic mapping procedure) and understanding the quality and the robustness of the matches. The goal of the present paper is to provide an overview of the methodological approach adopted in the GLAD NWG project to create the resulting mapping files, which serve as support for mapping and conversion activities commonly addressed by LCA practitioners. Additionally, this research aims to offer robust recommendations to the LCA community on how to adapt this mapping procedure to other nomenclature systems.

## 2 Materials and methods

The goal of the GLAD NWG project is to develop a common system to map the elementary flow lists in the nomenclature systems used by different background LCA databases connected to GLAD. This paper aims at describing the mapping procedure adopted in the context of the GLAD NWG and to summarize the main outcomes providing recommendations for the mapping activity of its application to existing LCA nomenclature systems and the potential links with subsequent activities.

The GLAD NWG produced the Methodology & Known Issues report (Vadenbo et al. 2022) to support and document the mapping activity and enhance transparency of the outcomes. The report also includes information about issues encountered and how those problems were addressed, with a primary focus on overcoming inconsistencies at the flow level rather than at the modeling or impact levels. The report describes the NWG’s decisions in addressing known issues. However, it should be noted that the report does not necessarily provide guidance for practitioners on how to address these concerns since users may prefer or need to resolve the issues using different approaches. Known issues addressed in the report include the mapping of the following:

- Contexts (e.g., issues related to find the correct correspondence between compartments for long-term missions, or the matching of overlapping contexts, or also the use proxy contexts)
- Flow names or “flowables” (e.g., matching of specific flows to a group of unspecific flows, or the matching of GHGs, or the matching of compounds vs. elements vs. ions, or use of proxy flow names)
- Flows (i.e., the pair flow names + context that do not match the criteria subsequently applied automatically, for example, by CAS or by name. This typically applies to water flows, land use flows, natural resources, and other types of flows with no CAS or synonyms associated

The terminology adopted by the NWG (“flow,” “context,” “flow name”) aligns to some extent with the ontology developed by the Big Open Network for Sustainability Assessment Information (BONSAI) for capturing data specifically relevant to life cycle sustainability assessment (Ghose et al. 2022). While in the BONSAI initiative, the term “flow” defines the measure of an entity that is produced or consumed regardless of its direction and whether the entity belongs to the technosphere or the biosphere (Weidema 2021), in the GLAD NWG, the same term refers to environmental flows (i.e., elementary flows) and includes information about the entity exchanged (“flow name”) and the compartment (“flow compartment”) which in turn may implicitly include the information on the flow direction (e.g., “emission to” denotes an output while “resource from” represents an input).

The NWG established a common data format and structure for the flow lists and mapping files. Additionally, the EC-Joint Research Centre (JRC) provided an ad hoc software tool known as the “GLAD Mapper Tool” (EC - Joint Research Centre 2022a) to support the project activities. Building upon these initial mapping resources, subsequent efforts were focused on three primary areas: (i) gathering and establishing the necessary data for running the Mapper Tool, along with expanding and fine-tuning its mapping algorithm, (ii) developing procedures and scripts for reviewing the preliminary mapping results, which were the “raw” outputs of the Mapper Tool, and (iii) documenting the approach, tools used, and common challenges encountered during the mapping activities.

The four reference flow lists mapped are (i) ecoinvent v3.7 (ecoinvent 2020), (ii) Environmental Footprint v3.0 (EC - Joint Research Centre 2022b), (iii) IDEA v2.3 (IDEA 2021), and (iv) U.S. Federal LCA Commons v1.0.3 (Edelen et al. 2019). Each of the four nomenclature systems is represented in the NWG by two members to guarantee a multilateral agreement on the approach to follow in the verification of results and the quality assessment. A flow in this context is a unique combination of flow name substances and contexts (see Table 1) and which is further defined by the units assigned to the flow (e.g., kg, MJ, and m^3^). It is worth mentioning that the update to the Environmental Footprint v3.1 in July 2022 does not affect the base flow list. Consequently, for the purpose of this work, the mapping results and outcomes related to Environmental Footprint v3.0 are fully extended to version 3.1.

Two factors that significantly affect the total length of a list are the number of flow names it contains and the number of compartments in which those flows are defined. Table 1 summarizes the main features in terms of the number of unique “flow names” (i.e., individual entries for resources, energy, land use, water, and other material flows that are exchangeable with the environment without considering the environmental compartment/sub-compartment), “compartments” (which describes the environmental context that exchanges the specific flow, including the sub-compartment and the direction of the exchange—e.g., resource/freshwater and emission to air/unspecified), and the total number of “flows” (as elementary exchanges), which is given by the flow name-context pairs provided in the native list.

Significant disparities are evident among the flow lists concerning the quantity of elementary flows, flow names, and compartments. Particularly noteworthy is the substantial discrepancy of three orders of magnitude in the number of total flows between the shortest list (IDEA) and the largest list (FEDEFL). In contrast, the disparity in number of unique flow names across the lists is less pronounced (i.e., only one order of magnitude); the ILCD-EF3.0 list contains the largest number of flow names, whereas IDEA features the smallest count. We can broadly categorize the four nomenclatures into two groups: ecoinvent and IDEA feature a relatively modest number of elementary flows, ranging from hundreds to thousands. In contrast, EF and FEDEFL extend into the order of tens of thousands. This discrepancy arises from the fact that ecoinvent and IDEA are databases that exclusively list elementary flows utilized within their respective datasets.

Conversely, ILCD-EF3.0 and FEDEFL aspire to serve as comprehensive platforms for elementary flows, encompassing all potential flow names, contexts, and combinations applicable to future datasets and impact assessment methods. It is worth noting that while ILCD-EF3.0 has a greater number of unique flow names, FEDEFL includes a significantly larger elementary flow list. The reason is that FEDEFL includes approximately three times as many sub-compartments as ILCD-EF3.0.

### 2.1 Common format and input file structure

Within the GLAD NWG approach, three files are required to obtain the resulting “mapped file”:

- The source flow list
- The target flow list
- The mapping criteria file

These three files also serve as inputs for the GLAD Mapper Tool to generate mapped files. The tool requires several pieces of information from the three input files, which must be provided in the proper format and structure. Specifically, the flow list files should include the following columns (Table 2).

For the proper interpretation by the Mapper Tool, the order of the fields must be respected (i.e., the fields shall be implemented by column in the spreadsheet, without blank columns and following the order reported in the table’s “column number”). The “Optional” fields refer to pieces of information that might be found in the original flow list but that are not required for this specific mapping activity; if the native flow list does not include this information, the column shall be left empty. Fields labeled as “Recommended” refer to those details that can be used by the Mapper Tool but that are not mandatory. The field “Other CAS” includes the list of CASs that can be found for one substance. It was populated using the URL-based API released by Common Chemistry (https://www.cas.org/services/commonchemistry-api). An ad hoc VBA function was developed to query the API via spreadsheet. The VBA function accepts in input either the name of a substance or a CAS number and, if the chemical is available in the database, it returns the list of CAS numbers available for that substance in the form of a string. This string is used to fill the “Other CAS” field for each substance in the GLAD flow lists. The four spreadsheets formatted for their operationalization in the NWG activity are publicly available in the GLAD NWG public repository (Global LCA Data Access 2021).

### 2.2 Format and structure of the mapping criteria files

In contrast to the flow lists, which are usually pre-existing files made available by LCA database developers, the mapping criteria file is a document that needs to be defined specifically for its mapping purpose; this hence represented a central task of the GLAD NWG in this project. The mapping criteria file allows for establishing patterns of correspondence between items of the list and allows the user to define exceptions. It is composed of different spreadsheets, each of which contains information on the correspondence between the source and target elementary flows. Table 3 summarizes the spreadsheet names and requirements and the type of information they bring.

The minimum requirement of the mapping criteria is the match of the flow context from the source to the target flow list. This means that the mapping criteria file shall contain at least the spreadsheet PROXY_CONTEXT_MATCH populated with the list of sub-compartments of the source flow list and the corresponding preferred sub-compartment in the target flow list. For example, when mapping the nomenclature of ILCD-EF3.0 to FEDEFL, the source sub-compartment “land use/occupation” in ILCD-EF3.0 corresponds to “resource/ground” in the FEDEFL system. Similarly, the ILCD-EF3.0 flows in the sub-compartment “emission/air/urban/high” corresponds to “emission/air/troposphere/urban/high” in FEDEFL. When a source elementary flow does not find a match of a sub-compartment in the target list, the Mapper Tool searches for a proxy sub-compartment among those listed in the spreadsheet “PROXY_CONTEXT_MATCH,” where the alternative target sub-compartment for the given source must be listed in the hierarchical order of preference. Proxy compartments too far from the source compartment shall be avoided. For example, the project team agreed that for the source compartment “emission/air/urban/ground level”, the target “emission/air/nonurban/low” is to be avoided; usually, the “unspecified” proxy closes the list of proxies (in other words, it is set as the last fall-back option unless the “unspecified” itself is the source context). In addition to matches for flows, flow names, and compartments, conversion factors are needed for given pairs of source-target flow names when either the units of the source and the target flow names differ or the defining properties of flow names differ between the lists (e.g., for energy carriers expressed by mass, 1 kg of coal with an energy content 15 MJ/kg is not the same as 1 kg of coal with an energy content of 20 MJ/kg).

As the mapping instruction is specific for each list pair, a different mapping criteria file must be defined for each of the 12 source–target combinations (available at https://github.com/UNEP-Economy-Division/GLAD-ElementaryFlowResources/tree/master/Mapping/Input/Mapping_files). It is important to highlight that, for a given flow list pair “list X and list Y,” the mapping instructions might change when switching from one matching direction to another. The GLAD Mapper must be executed in both directions by swapping the source–target lists and using one “mapping criteria” file for each direction. In the definition of the *from-X-to-Y* and *from-Y-to-X* correspondence, it is thus pivotal to enhance the consistency of the information in the bidirectional mapping criteria files. In this regard, detailed guidance for the mapping tool has been published (EC-Joint Research Centre 2022a).

**Fig. 1:**
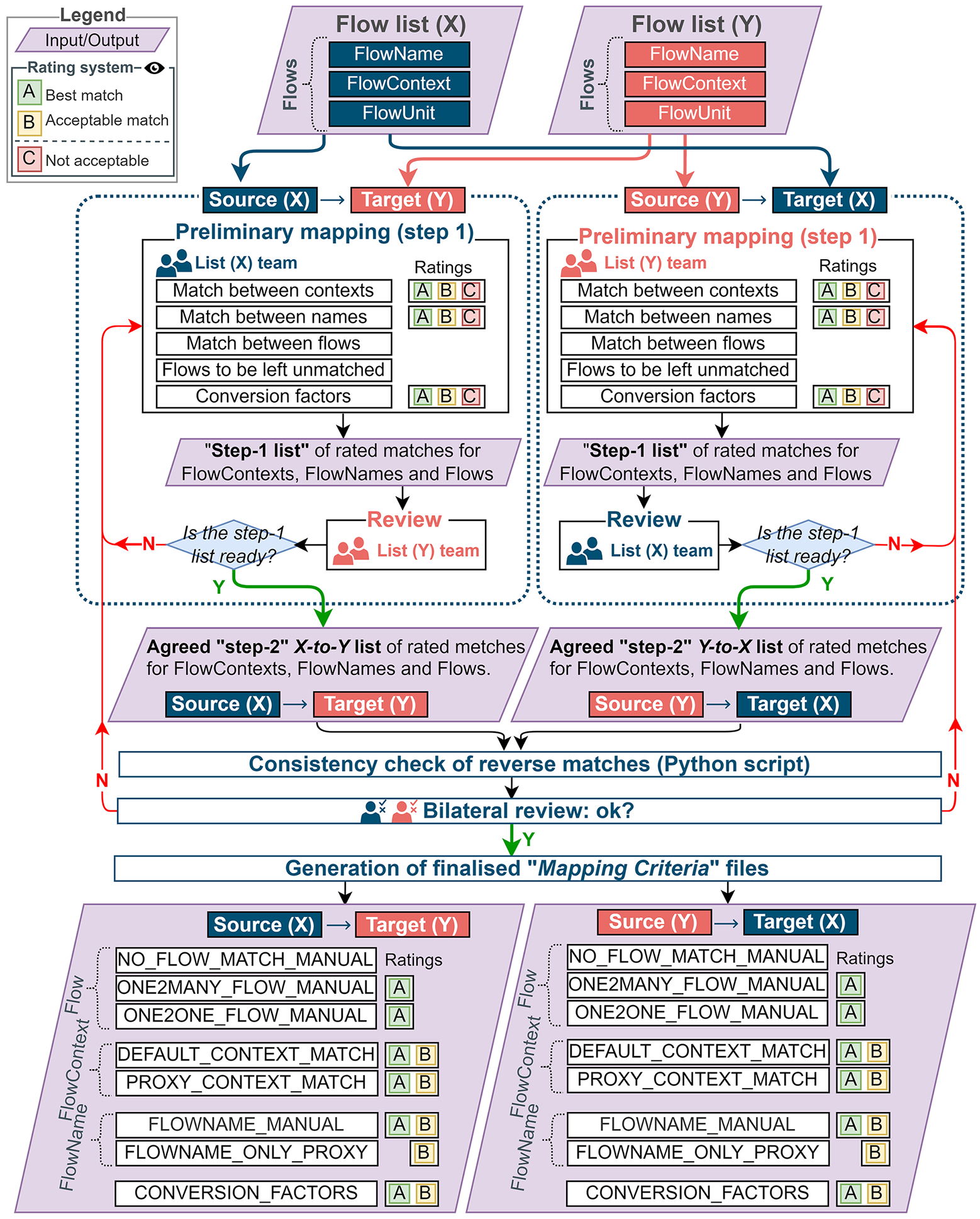
Workflow and main phases of the bilateral mapping activities as followed in the GLAD NWG to review to mapping criteria files of two generic flow lists “X” and “Y” in both directions (*from-X-to-Y* and *from-Y-to-X*)

Due to the data-intensiveness of the mapping activity described, one of the main challenges encountered in the definition of the mapping criteria file is to find the balance between the precision of the matches (representativeness) and the overall coverage (completeness). In this sense, the use of reasonable approximations (or “proxies”) to cover the source-side items increases the coverage of the mapping at expenses of precision. Despite potential subjective factors, to improve the transparency of the mapping procedure, NWG reviewers assigned quality scores (“A” for the best/ideal matches and “B” for acceptable matches, including “proxies”) to monitor the accuracy and precision of the matches. Owners of the participating lists *X* and *Y* review the proposed updates to the context mappings, and feedback from the entire NWG can also be requested at this stage. Figure 1 depicts the main steps taken by the NWG in the mapping procedure to define the mapping criteria files. The procedure is iterative and includes two review stages depicted in the figure: the first during the creation of the preliminary “source-target” mapping criteria file and the second to check the consistency when the mapping involves the same lists but in the opposite direction. In both reviews, to ensure the soundness of the matches, the owners of both source and target lists are directly involved.

To check the bidirectional consistency of mappings, a specific script taking the two mapping criteria files as input and returning an output of the form *from-X-to-Y* was implemented. This allowed to systematically test the agreement of *A* ratings on both sides and improve bidirectional consistency of the *from-X-to-Y* and the *from-Y-to-X* mappings. After the approval of the bilateral review, the mappings used to complete the mapping criteria files to then run the Mapper Tool to generate the mapped files for the pair of flow lists in the input.

In “flow name” mappings, flow names are grouped and matched in pairs to determine the most appropriate mapping result. This allows for flow name pairings, for example, of the main greenhouse gases CO_2_ and CH_4_ depending on the carbon source (i.e., fossil vs. biogenic/non-fossil) or from soil or biomass stock in the flow list where this distinction is made. The “flow name” mapping also applies to flow names that are broadly considered equivalent but are associated with different flow names, multiple CAS numbers, and chemical forms. It is important not to prefer proxies over best matches in contexts where both are available in the target list. This means structuring the flow name library to match the mapping sequence of the mapping script (prioritizing more specific flow names before proxy matches).

When establishing criteria for flow mapping, specific combinations of flow names and compartments are either matched, split, or blocked from being mapped. This procedure is designed to address flows, whether or not they meet the criteria, that are subsequently processed automatically by the mapping script to generate a preliminary mapping file. The primary groups of flows falling into this category are those related to land use (often with different names in flow lists and lacking CAS numbers or synonyms). It also applies to the exchange of water with the natural environment. Another scenario requiring manual flow-to-flow mapping is when natural resources are defined in flow lists as belonging to different environmental compartments. For example, the FEDEFL list categorizes natural gas as a resource in the air, whereas other lists consider natural gas an underground resource. These context combinations are not allowed according to the main context mapping. Therefore, certain exceptions to these rules must be made via flow-to-flow mappings, and these exceptions take precedence over the “default” approach.

### 2.3 Mapping criteria and their hierarchy

The GLAD Mapper is an IT tool developed within the GLAD NWG to assist in the creation of mapping files. Two main types of matches are performed by the tool, (i) “automatic matches,” executed by using the CAS number, flow name, list of synonyms, and other CASs as reported in the source and target flow lists, and (ii) “manual matches,” executed by interpreting the instructions as reported in the “mapping criteria” file. Table 4 summarizes the list of match types implemented in the tool algorithm.

For each combination of source and target lists, manual efforts are reduced to a minimum by following three steps: (i) compartments are mapped individually; further manual mapping is only applied to (ii) flow name groups that either lack attributes for unambiguous matching by the mapping script or are deemed particularly important and to (iii) flows (i.e., as unique combinations of flow names and compartments) when the general mapping criteria applied cannot be systematically implemented or otherwise fail (e.g., water- or land-related flows).

The matching algorithm is implemented in the tool by considering the level of confidence attributed to the different match types. In this regard, a higher priority is given to those match types having higher robustness that are under the user’s control. For example, the flow name “carbon dioxide” may match automatically with unwanted target flow names (e.g., by the CAS number with “carbon dioxide, biogenic” or by the synonym with “carbon dioxide, land use change” in a target flow list). Therefore, to prevent potential mismatches, the mapping tool includes a match priority scheme for the different elements of the flow lists. More generally, manual match types (i.e., defined by the user and indicated in the “match criteria” file) have a higher level of confidence (and thus a higher priority) than automatic matches.

**Fig. 2:**
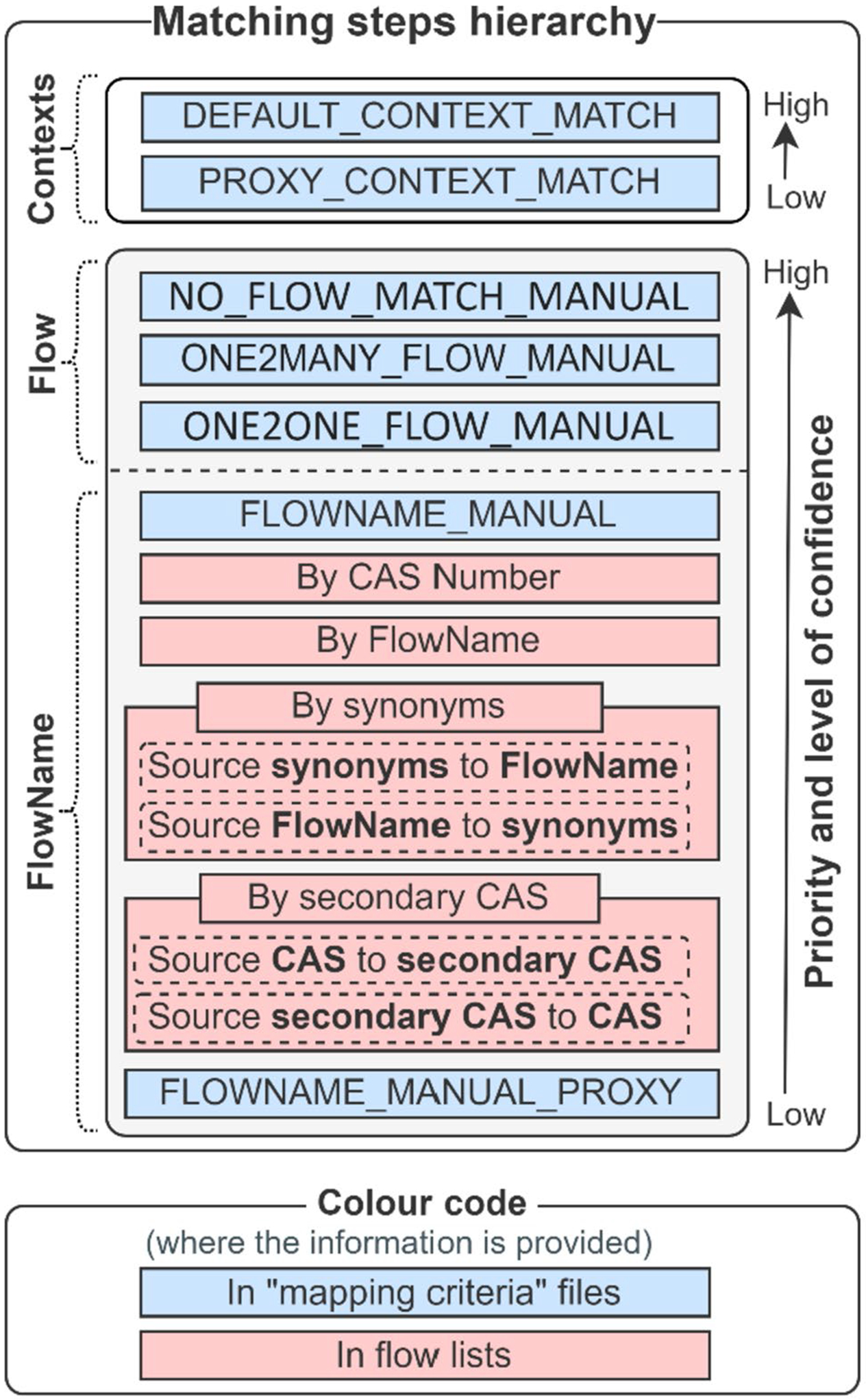
Hierarchy of match criteria (subdivided into criteria for *Flows, Contexts, FlowName*) implemented for the mapping of flows, flow names, and contexts

Figure 2 illustrates the prioritization implemented in the *GLAD Mapper Tool* for the different match types. The figure differentiates the match criteria by listing elements (flow name, contexts, and flows) ranked in descending order of priority. The match between contexts is performed in parallel (i.e., with the same priority) to that of flow names and flows; therefore, the prioritization of the context match is independent of that of the flows and flow names.

A higher priority is generally given to the match criteria defined in the “mapping criteria” file. The match type by “FLOWNAME_MANUAL_PROXY” is the only exception, which is considered the less preferable option (albeit still acceptable) to map a source flow name in the target list. This type of criteria might be defined when the list of target flows includes groups of generic substances (e.g., “hydrocarbons,” “pesticides,” and “insecticides”), and it should be understood as the last chance to find a match of the source flow in the target flows list; otherwise, it would remain “orphan” (i.e., without a match).

### 2.4 Overall mapping procedure

**Fig. 3:**
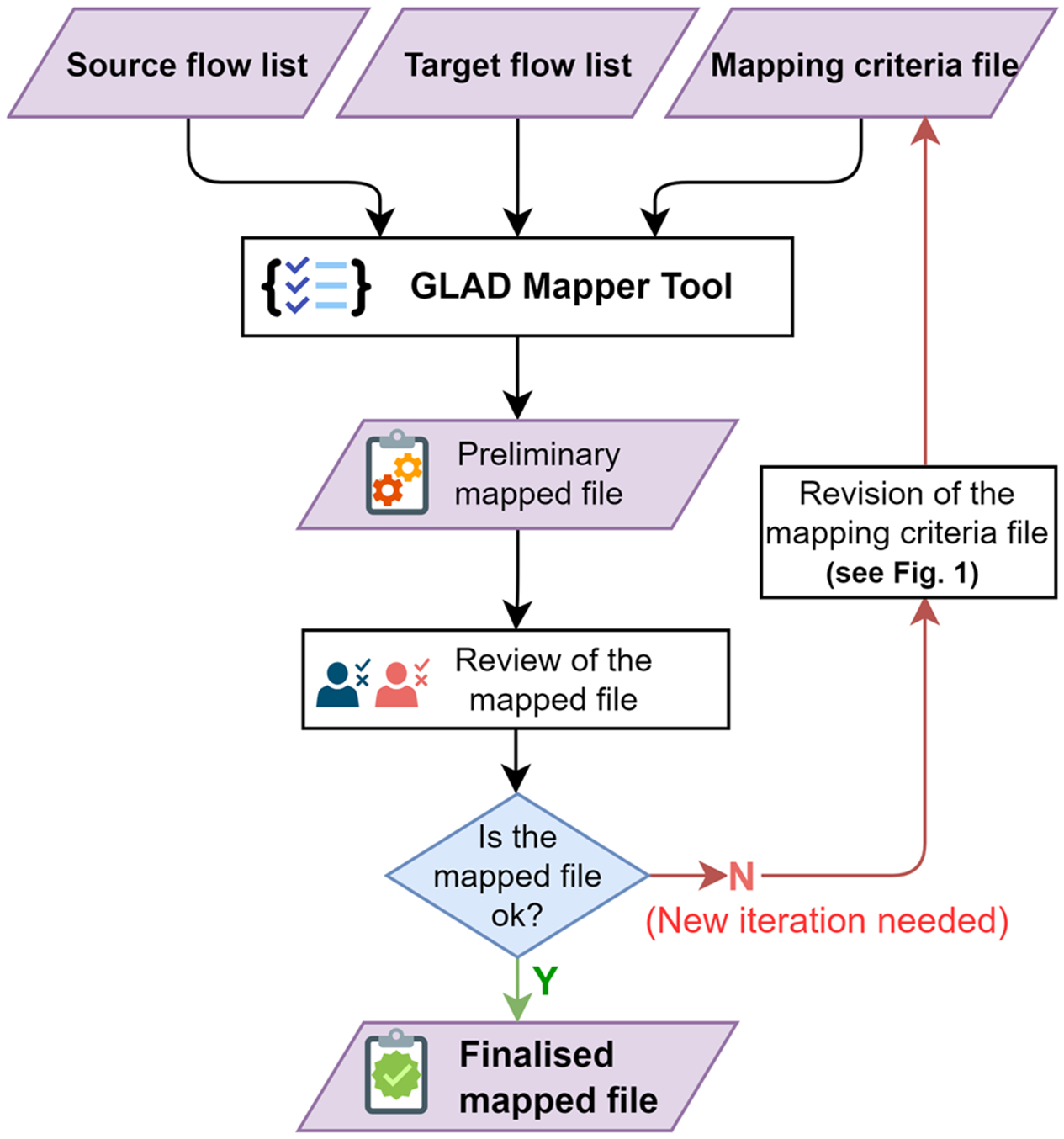
Overall mapping procedure to generate the mapped files

The overall mapping procedure involves different review phases, one at the level of the mapping criteria file definition (Fig. 1) and another bilateral review for the mapped file delivered. Figure 3 depicts the overall workflow for the generation of a single mapped file.

The resulting mapped file is automatically generated by the GLAD Mapper Tool; it contains pairwise flow attributes of the full source flow list and a corresponding item in the target list. This file represents thus a key element when it comes to converting an LCI provided in the source nomenclature into a specific target nomenclature system. Figure S1 details the logic of the mapper algorithm implemented in the Mapper Tool to classify the correspondences with the generic *ith* elementary flow of the source flow list. Table 5 details the fields included in the mapped file and the type of content delivered that can help improve or interpret data exchange format conversion.

Notably, the mapped files contain both those details needed for the conversion (UUIDs, context, flow name names, and conversion factors) and, for the sake of transparency, additional details about the quality of each correspondence, namely, on the confidence level that is attributed bilaterally to the mapping of context, flows, and conversion factors during the definition of the mapping criteria files (Fig. 1). The 12 finalized mapped files with mappings between each list are made available in GLAD’s GitHub repository (https://github.com/UNEP-Economy-Division/GLAD-ElementaryFlowResources/tree/master/Mapping/Input/Mapping_files), in which all changes to the mapping resources are recorded, including the version control of all files.

Table S1 summarizes the absolute number of flows matched by type for each of the 12 mapped source–target list combinations achieved after the last iteration. The table shows that a significant portion of mapped flows are matched automatically by CAS or assigned manually by flow name matches. The high rate of selected proxy flows is linked to assigning more specific flows in in the source flow list with more general flow families (see FLOWNAME_MANUAL_PROXY criteria description in Table 4). This highlights the need for particular attention to these fields when compiling mapping criteria. The coverage achieved for the combination is calculated as the ratio of the number of source flows matched (*D*) to the total number of elementary flows in the source list (*A*). This ratio can be further subdivided into coverage associated with “best matches” only (*C*) and coverage related to “proxy matches” only (*B*). These ratios (coverages) provide an indication of the capability of the target list to represent the source list.

## 3 Results and discussion

The GLAD mapping activity has valuable implications for the LCA field. By mapping data across four major nomenclature systems, the creation of a solid foundation for improving data accessibility, compatibility, and reliability in LCA studies is possible. The output of this activity supports practitioners to convert LCI data between different systems, enabling more thorough analyses. The achievement of the outcomes was possible through the iterative procedure described in the previous sections. To reach a satisfactory balance between the completeness and accuracy of the matches within the NWG, seven iterations were needed. Table S2 and Fig. S2 in SM summarize the actions performed at each iteration and of the resulting mapping coverage, both in terms of “best” matches and in terms of “other” matches (i.e., using a proxy compartment or a proxy flow). Table S2 also shows the percentage of best matches considered in all the elementary flows matched at each iteration and the percentage of “no match” relative to the 1.31 million elementary flows (i.e., the total number of elementary flows in the target side over the 12 combinations). In addition, Fig. S2 shows the evolution of the level of overall coverage (i.e., considering the source elements to be mapped for the 12 source–target combinations jointly) that was reached after each iteration. Figure S3 details the coverage evolution by iteration and by source–target combination.

Notably, the implementation of the “FLOWNAME_MANUAL_PROXY” match type (see Table 4 for further details on match types), put in place at iteration number six, led to a significant increase in the coverage, which in relative terms (Fig. S3) was found to be more pronounced in the shorter flow lists (IDEA and ecoinvent). However, it also must be noted that the increase in coverage for this implementation is mostly associated with proxies (generic “hydrocarbons” and “pesticides”) rather than more specific “best matches.” The percentage of “best matches” decreased in fact from 83.0 to 58.4% after the 6th iteration in contrast to the overall percentage of coverage, which increased from 32.2 to 43.9%. Considering this, we suggest including the match type “FLOWNAME_MANUAL_PROXY” when the specific circumstance allows assuming a lower level of accuracy, which is still acceptable, in return for reducing the number of orphan items in the source flow list or LCI. On the other hand, the addition of a “secondary CAS” in all the flow lists (5th iteration) allowed for the highest increase (in absolute terms) of “best matches,” which was particularly remarkable when starting from the largest flow list (i.e., FEDEFL, as Fig. S3 shows). In this regard, we recommend including secondary or alternative CASs associated with chemical substances in both the source and target flow lists.

The main result of the mapping procedure is summarized by the coverage achieved across the final 12 source–target combinations of mapped fields (available at https://github.com/UNEP-Economy-Division/GLAD-ElementaryFlowResources/tree/master/Mapping/Output/Mapped_files). A possible implementation for applying the resulting mappings is the *Lavoisier* library (Savioli et al. 2023), a Python library for converting LCA inventory datasets between different data formats. *Lavoisier* aims to provide a more cohesive conversion process with minimal loss of information compared to the previously available *openLCA Converter* (GreenDelta GmbH 2015). It currently supports conversion between ecoSpold2 and ILCD formats and plans to expand to other versions and dataset types in the future. *Lavoisier* is already used in the Brazilian Life Cycle Inventory database (IBICT 2016) and addresses some shortcomings of the *openLCA Converter*, such as preserving Pedigree matrix uncertainty, coefficient information, input flows, parameters, and mathematical equations. *Lavoisier* already supports an automated mapping between ecoinvent 3.7 and EF 3.0 nomenclatures and it could be extended to support nomenclature conversions based on the results from the work presented in this paper, ideally in a way that allows adding new or updated mappings at runtime. This is crucial aspect when it comes to maintaining the nomenclature mappings independently from the conversion software’s source code and thus enable fast availability of an updated mapping service when a new version of a given nomenclature system is published. Such a system, combining the mapping results generated by the presented framework with a software-automated mapping facility, would enable transparent automated conversions between various supported data formats and nomenclature systems. This integration could extend to software tools and systems like GLAD, making the process of converting between different data formats and nomenclature systems not only more user-friendly but also significantly reducing the workload and potential errors for LCA practitioners. Additionally, it could perform these conversions transparently in the background, eliminating the need for explicit user interaction.

Regarding the outcomes of the mapping activity in this work, the level of coverage of the 12 mapping combinations achieved after the last iteration is summarized in Table 6. Table S1 provides the underlying absolute values distinguished by match type). The percentages are expressed as the number of mapped flows (matches) relative to the number of flows in the source list. The information is provided both in terms of total matches (i.e., considering both acceptable matches, including proxy sub-compartments/flow names, and “best matches”). The rows may be read as the “capability of covering” the target list, while the columns may express the “capability of being covered” of the source list.

Higher levels of coverage are achieved with the two larger flow lists (ILCD-EF v3.0 and FEDEFL v1.0.3) on the target side. These flow lists show a higher capability of covering other flow lists provided in other nomenclature systems. The coverage capability of a target flow list in terms of both total matches and “best matches” appears more closely linked to the number of available unique flow names than to the number of sub-compartments. This is reasonable given that most lists considered include “unspecified” or more aggregated sub-compartments (in the case of FEDEFL), which can serve as proxies for specific sub-compartments. However, despite the high number of iterations performed in the NWG work, the full coverage of a source flow list was not possible in any of the source–target combinations. It should be highlighted that the shorter lists (i.e., ecoinvent and IDEA) showed a higher capability of being covered. In other words, an LCI provided in these nomenclatures are—on average—easier to be matched. It must be noted that a perfect conversion (i.e., without a loss of information) is rarely possible in practice. In this regard, a trade-off is necessary between maximizing completeness and ensuring a high degree of accuracy (flow correspondence) for each item matched when establishing mapped files.

Overall, the different flow lists that are used in life cycle assessment represent not only different naming conventions or units for flows but also, to some extent, fundamentally different ways of defining or characterizing the interactions or interventions of humans with the natural environment. Some flow lists might focus on short- or mid-term environmental impacts of a product or process, while others take a more comprehensive, long-term view. Other fundamental differences can be related to inclusion or not of the distinction of fossil and biogenic carbon emissions. While establishing a common and widely recognized reference flow list would be a significant step towards harmonizing the nomenclature used in the LCA community, it may not be possible to achieve the necessary level of consensus in the short term. One reason is that elementary flows from database providers such as ecoinvent or IDEA are closely linked to the raw data each database uses to generate its datasets. Forcing a specific nomenclature system could cause deviations in the elementary flow from the raw data source. However, the pairwise mapping applied in the current study allows for a neutral examination of these differences, providing insights that can serve as a foundation for future harmonization efforts.

## 4 Conclusions and outlook

The work carried out demonstrated the applicability of the mapping approach defined within the GLAD NWG. The mapping framework has been applied to four existing nomenclature systems broadly used in the LCA community. The activity performed within the NWG produced guidance and materials enabling the extension of the mapping to other nomenclature systems. As the main results of this project, a high level of coverage is found to be mainly linked to the availability of unique flow names and, to a lesser extent, to the granularity of the environmental compartments. However, under the mapping framework applied in the NWG, full coverage was not achieved by any of the four flow lists, despite the use of proxies both at the flow name level and the sub-context to increase the level of coverage. In this regard, while mapping is not a novel concept, it is essential to emphasize that the approach described in this work stands apart from previous efforts. Unlike proprietary mapping conducted by software providers, the GLAD approach is open and transparent. This key distinction fosters transparency in the mapping process and holds the potential for greater interoperability across the entire community, underlining the NWG commitment to openness and collaboration. Furthermore, the mapping activities performed in this work may represent a starting point towards the definition of a common central hub for mapping LCIA methods and datasets further facilitating the interoperability of common data. Enhanced data accessibility and interoperability will benefit the whole community and the mainstream applicability of LCA and is the foundation for key sustainability initiatives. Policymakers rely on it, e.g., for the development of sound policies. Industries will be able to base their innovation and strategic sustainability decisions on more robust information. In this sense, under the umbrella of the UNEP Life Cycle Initiative, it is important to ensure the involvement of stakeholders such as LCA software providers, LCIA method developers, and LCI data providers.

In conclusion, the mapping results of this study are relevant to several widely used nomenclature systems. The 12 resulting mapped files represent a valuable instrument for LCA practitioners. For example, they can use these files to translate process data from one of the four nomenclature systems covered in this study to another. The implementation of the resulting mappings through the *Lavoisier* library offers a promising solution for efficient and accurate conversion of LCA inventory datasets across various data formats and nomenclature systems. This approach addresses limitations of existing converters, provides flexibility for future updates and maintenance, and has the potential to streamline the conversion process for LCA practitioners, making it more user-friendly and less error-prone, even enabling background automation in tools like GLAD. Furthermore, the *GLAD Mapper Tool* developed by the NWG may serve as a valid support for performing mappings between other flow lists. Overall, the outputs of the NWG activities are key inputs for improving the features of the GLAD system, which allows for the conversion of LCI data from one nomenclature to another. Therefore, the ongoing maintenance of flow lists, mapping criteria files, and mapped lists, as well as the expansion of bidirectional mapping to other nomenclature systems, would be beneficial to the LCA community.

## Supplementary Material

Supplementary Information

## Figures and Tables

**Table 1:** Main elements and structure of the four nomenclature systems included in this study

Flow list	Abbreviation	Unique flow names	Compartments	Total flows
ecoinvent v3.7	ecoinvent	1404	22	4310
Environmental Footprint v3.0/3.1	ILCD-EF3.0	7741	36	93,993
IDEA v2.3	IDEA	612	20	903
U.S. Federal LCA Commons v1.0.3	FEDEFL	5933	114	278,602

**Table 2:** Format specifications for the flow list files to feed the GLAD Mapper (semicolon character is used as the list separator)

(Column number) column name	Requirement	Example of content
(1) FlowName	Mandatory	Levomenthol
(2) CASNumber	Recommended	2216-51-5
(3) Formula	Optional	C_10_H_20_O
(4) Synonyms	Recommended	(1R,2S,5R)-(−)-menthol; cyclohexanol, 5-methyl-2-(1-methylethyl)-, (1R,2S,5R)-
(5) FlowUnit	Mandatory	kg
(6) Class	Optional	Chemicals
(7) ExternalReference	Optional	https://sor.epa.gov/
(8) Preferred	Optional	1
(9) FlowContext	Mandatory	Emission/air/troposphere/rural/ground-level
(10) FlowUUID	Mandatory	fec90d1d-f3f4-3ede-846b-9dff48e20c25
(11) AltFlowUnit	Optional	-
(12) AltUnitConversion Factor	Optional	-
(13) OtherCAS	Recommended	114376-98-6; 89-78-1; 2216-51-5

**Table 3:** Contents and specifications for the mapping criteria file

Spreadsheet name	Requirement	Type of content listed
NO_FLOW_MATCH_MANUAL	Optional	Source elementary flows (flow name + context) not to be matched
ONE2MANY_FLOW_MANUAL	Optional	Source elementary flows (EFs) and corresponding target EFs to be matched
ONE2ONE_FLOW_MANUAL	Optional	Source EFs and corresponding target EF to be matched
FLOWNAME_MANUAL	Recommended	Source flow name and corresponding target flow name to be matched
FLOWNAME_MANUAL_PROXY	Optional	Source flow name and corresponding target flow name to be matched
DEFAULT_CONTEXT_MATCH	Mandatory	Source sub-compartments and corresponding target sub-compartments
PROXY_CONTEXT_MATCH	Recommended	Source sub-compartments and corresponding target sub-compartments
CONVERSION	Recommended	Source-target flow names and units and corresponding conversion factor to express the given source flow in the unit of the target flow

**Table 4:** Match types for sub-compartments, flow name, and elementary flows implemented in the mapping tool

Match type label	Match type description	Note
NO_FLOW_MATCH_MANUAL	The source elementary flow is not mapped in the target list to prevent it from being considered by the tool and potentially leading to incorrect mapping entries	Elementary flows are required in case a specific flow should not be matched for specific compartments but can find matches in other proxy compartments
ONE2MANY_FLOW_MANUAL	The source elementary flow corresponds to at least two flows in the target list	In this project, it was used for IDEA land transformation flows which include “from” and “to” in one single flow, while in other systems, “from” and “to” are listed as two separate flows
ONE2ONE_FLOW_MANUAL	The elementary source flow corresponds to one single flow in the target list	Groups of flows that do not match with automatic criteria (e.g., by CAS, name or synonyms) or for which the criteria can lead to mismatches (e.g., carbon dioxide having the same CAS irrespective of list-specific differentiations based on the source of the carbon, i.e., biogenic, fossil, etc.)
FLOWNAME_MANUAL	Only flow names (flow name) in the source and target flow lists are considered	The match of the context is resolved by DEFAULT_CONTEXT_MATCH or by PROXY_CONTEXT_MATCH. The source side shall include each flow only once, while the target list can contain multiple entries for the same flow
FLOWNAME_MANUAL_PROXY	Applied when a better proxy is not captured by other iterations, e.g., via automatic CAS match or FLOWNAME_MANUAL (Low-rank matches)	It is only applied after the other checks and linking steps in the mapping script. It is meant for matches that have a lower quality rank, e.g., specific substances to a group of substances (for example a specific chemical to “pesticides unspecified” or a specific hydrocarbon to “hydrocarbons unspecified”)
DEFAULT_CONTEXT_MATCH	Match of default or primary proxy of compartments/sub-compartments (flow contexts) for a given combination of source and target lists	This table establishes the exact match or the best proxy available for each entry from the source to the target list
PROXY_CONTEXT_MATCH	Specification of secondary context proxies, where applicable	It allows for more than one suitable or relevant context in the target list to be considered by the mapping script. Secondary context proxies are sorted hierarchically according to proximity to the source list
CAS	Identical source and target CAS number	Match automatically executed
NAME	Identical source and target flow name	Match automatically executed
SYNONYM_TO_NAME	One of the synonyms of the source matches one of the names of the target	Match automatically executed
NAME_TO_SYNONYM	The name of the source matches one of the synonyms of the target	Match automatically executed
SECOND_CAS	Either the main CAS or one of the secondary CAS matches with either the primary or one of the secondary CAS in the target (main to main CAS is already resolved in the “CAS” case, thus is excluded from this check)	Match automatically executed

**Table 5:** Fields and type of content of the mapped file generated by the GLAD Mapper Tool. See Table 3 in Vadenbo et al. (2022)

Column name in the mapped file	Example of the content
SourceListName	IDEAv2.3
SourceFlowName	Natural gas liquids, 46.5 MJ/kg
SourceFlowUUID	56991ece-e1c9-440f-96b0-0ca25b69d6c9
SourceFlowContext	Resources/ground/non-renewable energy resources
SourceUnit	kg
ConversionFactor	0.995423194^b^
MapType	FLOWNAME_MANUAL
TargetListName	Ecoinvent_3.7
TargetFlowName	Gas, natural, in ground ^c^
TargetFlowUUID	7c337428-fb1b-45c7-bbb2-2ee4d29e17ba
TargetFlowContext	Natural resource/in ground
TargetUnit	m^3^
Mapper	(Full name 1)
Verifier	(Full name 2)
LastUpdated	29/09/2022
FlowName condition (S)^a^	~^d^
FlowName condition (T)^a^	~^d^
FlowName confidence (S)^a^	B
FlowName confidence (T)^a^	B
Context condition (S)^a^	~^d^
Context condition (T)^a^	~^d^
Context confidence (S)^a^	B
Context confidence (T)^a^	B
Conversion confidence (S)^a^	B
Conversion confidence (T)^a^	B

a Rates for confidence level and condition of flow names, contexts, and conversion factors have been added to the mapped files generated by the tool

b To express the source flow in the unit of the target flow

c The LHV of “gas, natural, in ground” considered in ecoinvent 3.7 is 46.7138 MJ/kg

d “Condition” indicates if the scope of the source element (i.e., the flow name or context) is broader (“>”), smaller (“<”), similar (“~”), equal (“=”) or different (“< >”) to the scope of the matched target element

**Table 6:** Flow list coverages in the finalized mapping files. Within brackets, the coverage considers “best matches” only

Target list	Source list			
	ecoinvent v3.7	ILCD-EF v3.0	IDEA v2.3	FEDEFL v1.0.3
ecoinvent v3.7	-	23.5% (4.9%)	68.0% (35.9%)	28.8% (7.2%)
ILCD-EF v3.0	98.6% (95.8%)	-	95.0% (89.2%)	89.4% (68.7%)
IDEA v2.3	41.2% (20.3%)	17.3% (1.1%)	-	20.7% (4.7%)
FEDEFL v1.0.3	94.5% (81.5%)	62.3% (52.5%)	90.3% (86.5%)	-

## Data Availability

The elementary flow lists mentioned in Table 1, the 12 mapping criteria files, the Glad Mapper Tool, and the 12 resulting mapping file of this work are publicly available in the GitHub of UNEP GLAD at the following url: https://github.com/UNEP-Economy-Division/GLAD-ElementaryFlowResources/.
