# Supplementary material for: Elementary flow mapping across life cycle inventory data systems: A case study for data interoperability under the Global Life Cycle Assessment Data Access (GLAD) initiative
Source: Int J Life Cycle Assess. Author manuscript; Available in PMC 2024 Dec 30. (PMC11684518; doi:10.1007/s11367-024-02286-x)
Supplement: Supplementary Information [file NIHMS2021963-supplement-Supplementary_Information.docx]

# Elementary flow mapping across life cycle inventory data systems: a case study for data interoperability under the Global Life Cycle Assessment Data Access (GLAD) initiative

**Antonio Valente ^1*^, Carl Vadenbo ^2^, Simone Fazio ^1^, Koichi Shobatake ^3^, Ashley Edelen ^4^, Thomas Sonderegger ^2^, Selim Karkour ^3^, Oliver Kusche ^5^, Edward Diaconu ^1^, Wesley W. Ingwersen ^6^**

^1^ European Commission, Joint Research Centre, Directorate D – Sustainable Resources, 21027 Ispra (VA), Italy

^2^ ecoinvent Association, Technoparkstrasse 1, 8005 Zürich, Switerland

^3^ TCO2 Co. Ltd., 6F Daigonagamori Bldg., 12 Nandocho, Shinjuku-ku, Tokyo 162-0837, Japan

^4^ Eastern Research Group (ERG), Cincinnati, OH 45219, U.S.A.

^5^ Oliver Kusche Research & Consulting, Marie-Curie-Str. 1, 79104 Freiburg, Germany.

^6^ U.S. Environmental Protection Agency, Center for Environmental Solutions and Emergency Response, Washington, DC U.S.A.

**Corresponding author current address: valente@ecoinvent.org*

# Supplementary Materials

Supplementary materials include the representation of the GLAD Mapper Tool algorithm used to generate the resulting mapped files (Figure S1). These mapped files are provided in the form of worksheets accessible through the GitHub repository dedicated to the Nomenclature working group at this link https://github.com/UNEP-Economy-Division/GLAD-ElementaryFlowResources/tree/master/Mapping/Output/Mapped_files. Furthermore, in Table S1, we present the absolute underlying values used to calculate the relative coverage reached after the last iteration for each source-target flow list combination.

Finally, we provide details about the outcomes after each of the seven iterations during which the representatives of the source and target flow lists reviewed the mapping criteria files. In this regard, Table S2 summarizes the main aspects and actions reviewed during each iteration and their effects in terms of coverage. Figure S2 depicts the evolution of "best matches" and "other matches" over the seven iterations for the overall set of flows to be mapped, regardless of the flow list. Meanwhile, Figure S3 provides the evolution of coverage subdivided by flow list combination.


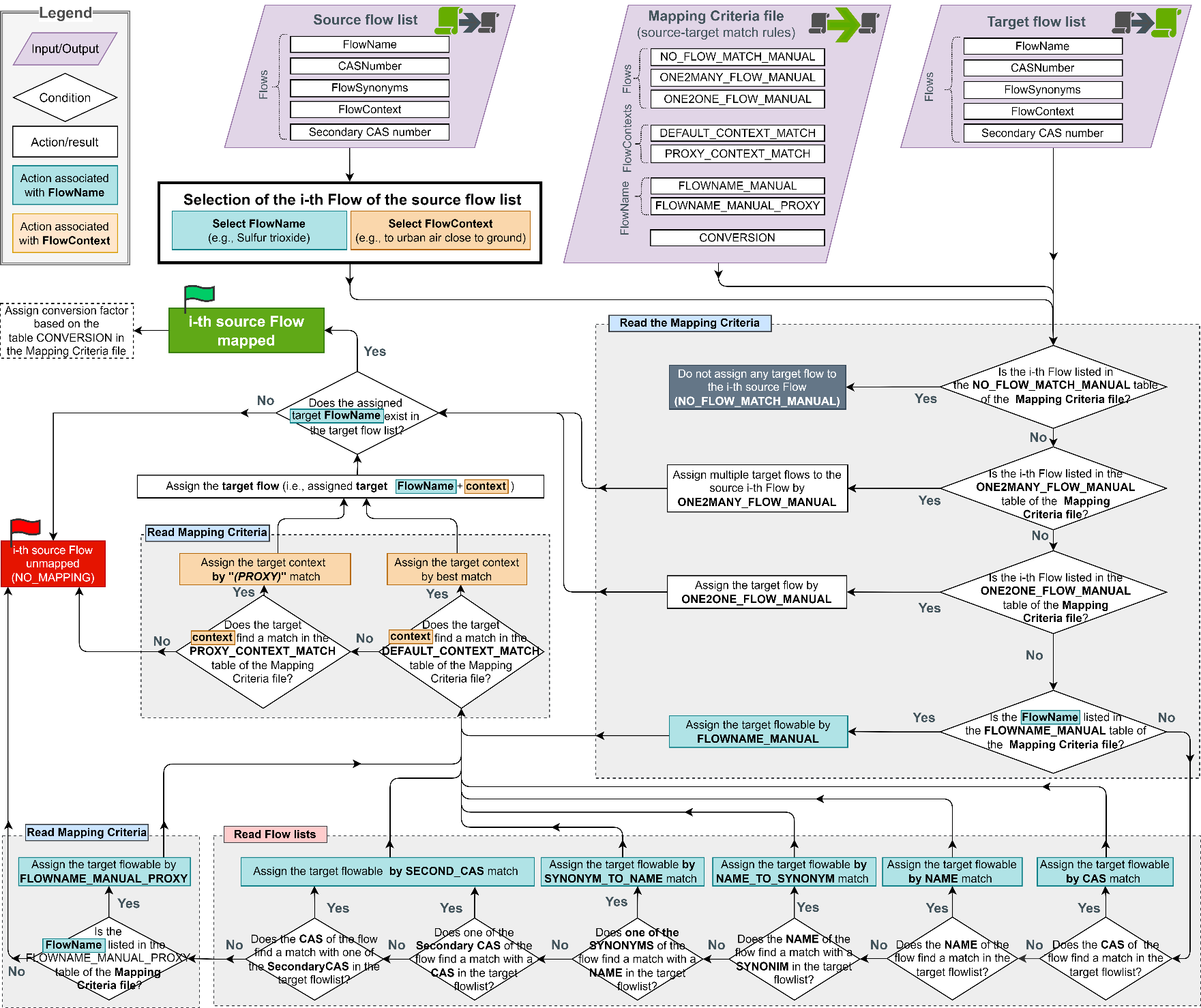


**Figure S1**. Flow diagram showing the logic of the *GLAD Mapper* algorithm for the mapping of a generic *i-th flow* of the source flow lists.

**Table S1:** Summary of the number of flows matched by types per each of the 12 finalised mapped source-target list combinations. The suffix “*(PROXY)*” refers to flows that match with a proxy context.

| **Match type** | ***Source list*** | *EF3.0* | *EF3.0* | *EF3.0* | *IDEA* | *IDEA* | *IDEA* | *FEDEFL* | *FEDEFL* | *FEDEFL* | *ecoinvent* | *ecoinvent* | *ecoinvent* |
| --- | --- | --- | --- | --- | --- | --- | --- | --- | --- | --- | --- | --- | --- |
|  | ***Target list*** | *FEDEFL* | *ecoinvent* | *IDEA* | *FEDEFL* | *ecoinvent* | *EF3.0* | *ecoinvent* | *IDEA* | *EF3.0* | *FEDEFL* | *IDEA* | *EF3.0* |
| NO_FLOW_MATCH_MANUAL | | 21 | 10 | 33 | 3 | 4 | 3 | 87 | 318 | 85 | 6 | 26 | 10 |
| ONE2MANY_FLOW_MANUAL | | 0 | 0 | 0 | 0 | 20 | 20 | 0 | 0 | 0 | 0 | 0 | 0 |
| ONE2ONE_FLOW_MANUAL | | 117 | 268 | 86 | 26 | 23 | 25 | 181 | 122 | 228 | 79 | 59 | 218 |
| FLOWNAME_MANUAL | | 3 116 | 1 887 | 315 | 175 | 157 | 164 | 9 016 | 4 156 | 10 918 | 1 241 | 322 | 1 406 |
| FLOWNAME_MANUAL (*PROXY*) | | 20 | 161 | 799 | 2 | 11 | 18 | 1 268 | 2 546 | 3 237 | 219 | 243 | 81 |
| CAS | | 45 401 | 2 337 | 593 | 574 | 124 | 601 | 10 196 | 7 827 | 178 155 | 2 144 | 465 | 2 489 |
| CAS (*PROXY*) | | 5 | 623 | 1 216 | 0 | 87 | 1 | 1 054 | 1 548 | 21 035 | 131 | 108 | 6 |
| NAME | | 235 | 31 | 8 | 4 | 0 | 2 | 119 | 131 | 483 | 19 | 0 | 11 |
| NAME (*PROXY*) | | 2 | 242 | 722 | 0 | 0 | 0 | 787 | 2 266 | 20 766 | 232 | 70 | 3 |
| SYNONYM_TO_NAME | | 0 | 0 | 0 | 0 | 0 | 0 | 3 | 0 | 49 | 0 | 0 | 10 |
| SYNONYM_TO_NAME (*PROXY*) | | 0 | 0 | 0 | 0 | 0 | 0 | 0 | 0 | 0 | 0 | 0 | 0 |
| NAME_TO_SYNONYM | | 0 | 9 | 0 | 0 | 0 | 0 | 0 | 0 | 0 | 0 | 0 | 0 |
| NAME_TO_SYNONYM (*PROXY*) | | 0 | 0 | 0 | 0 | 0 | 0 | 0 | 0 | 0 | 0 | 0 | 0 |
| SECOND _CAS | | 513 | 97 | 21 | 2 | 1 | 2 | 515 | 681 | 1 675 | 5 | 8 | 2 |
| SECOND_CAS (*PROXY*) | | 0 | 24 | 67 | 0 | 0 | 0 | 121 | 341 | 479 | 1 | 4 | 0 |
| FLOWNAME_MANUAL_PROXY | | 9 205 | 14 035 | 2 772 | 35 | 125 | 37 | 49 912 | 19 552 | 6 676 | 6 | 362 | 42 |
| FLOWNAME_MANUAL_PROXY (*PROXY*) | | 0 | 2 399 | 9 665 | 0 | 76 | 0 | 7 052 | 18 662 | 5 538 | 0 | 145 | 0 |
| NO_MAPPING (orphan source flows) | | 35 379 | 71 889 | 77 729 | 85 | 289 | 43 | 198 378 | 220 770 | 29 363 | 233 | 2 524 | 52 |
| **A: TOT source flows matched** | | 58 614 | 22 113 | 16 264 | 818 | 624 | 870 | 80 224 | 57 832 | 249 239 | 4 077 | 1 786 | 4 268 |
| **B: TOT PROXY matches** | | 9 232 | 17 484 | 15 241 | 37 | 299 | 56 | 60 194 | 44 915 | 57 731 | 589 | 932 | 132 |
| **C: TOT best matches** | | 49 403 | 4 639 | 1 056 | 784 | 329 | 817 | 20 117 | 13 235 | 191 593 | 3 494 | 880 | 4 146 |
| **D: TOT flows in the source list** | | 93 993 | 93 993 | 93 993 | 903 | 903 | 903 | 278 602 | 278 602 | 278 602 | 4 310 | 4 310 | 4 310 |
| **Overall coverage (A/D)*100** | | **62.3%** | **23.5%** | **17.3%** | **90.3%** | **68.0%** | **95.0%** | **28.8%** | **20.7%** | **89.4%** | **94.5%** | **41.2%** | **98.6%** |
| **Coverage best matches only (C/D)*100** | | **52.5%** | **4.9%** | **1.1%** | **86.5%** | **35.9%** | **89.2%** | **7.2%** | **4.7%** | **68.7%** | **81.5%** | **20.3%** | **95.8%** |

**Table S2:** Actions/improvements involved in each mapping iteration and their effects in terms of variation of overall coverage. Differences in “best”, “other” and “no” matches are expressed with respect to the previous iteration.

| **Iteration** | **Improvements/actions** | **Difference in “Best matches”** | **Difference in “Other matches”** | **Difference in “No matches”** | **% “Best matches”** | **% Mapped** |
| --- | --- | --- | --- | --- | --- | --- |
| 1 | Mapping criteria file generation | 0 | 0 | 0 | 84.3% | 31.1% |
| 2 | i) Fix wrong context names (IDEA) ii) Fine-tuning of mapping criteria | +392 | +5 623 | -6 015 | 83.0% | 31.6% |
| 3 | i) Adjustment of CAS format (EFv3.0)  ii) Addition of missing CAS (EFv3.0)  iii) Bidirectional review mapping criteria | +753 | +327 | -1 038 | 83.0% | 31.7% |
| 4 | i) Adjustment of CAS format and characters (FEDEFL)  ii) Added some missing CAS in the ecoinvent list  iii) Bidirectional review mapping criteria | +569 | +339 | -885 | 82.9% | 31.8% |
| 5 | i) Added secondary CAS (all flow lists)  ii) Tuning of mapping criteria (bidirectional) | +4255 | +691 | -4949 | 83.0% | 32.2% |
| 6 | i) FLOWNAME_MANUAL_PROXY match type for pesticides and hydrocarbon  ii) Bidirectional review mapping criteria | -13 006 | +144 628 | -131 651 | 58.4% | 43.9% |
| 7 | i) Removed inconsistent water flows mappings  ii) Addition of pesticides-specific flows and generic ones for other compartments  iii) Bidirectional review mapping criteria | +244 | +27 | +383 | 58.4% | 43.9% |


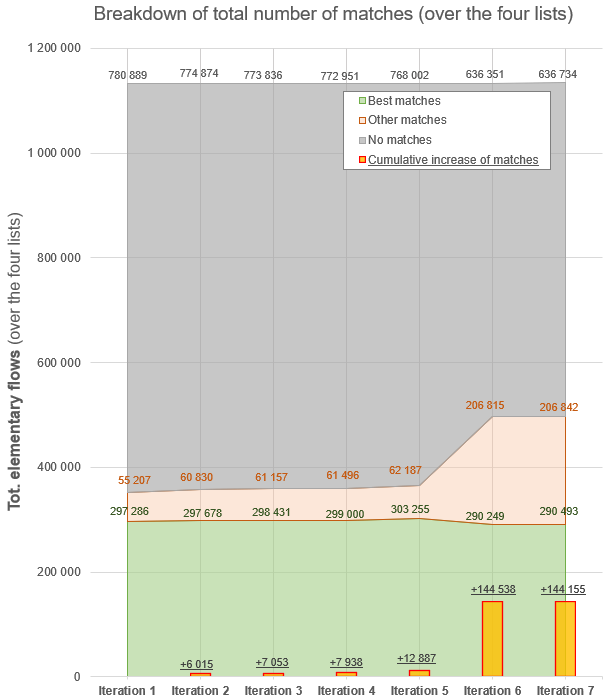


**Figure S2**: Evolution of overall coverage at each the seven iterations. Orange bars represent the absolute increase of total matches (i.e., regardless of whether “best” or “other” match type) cumulative with respect to the first iteration.


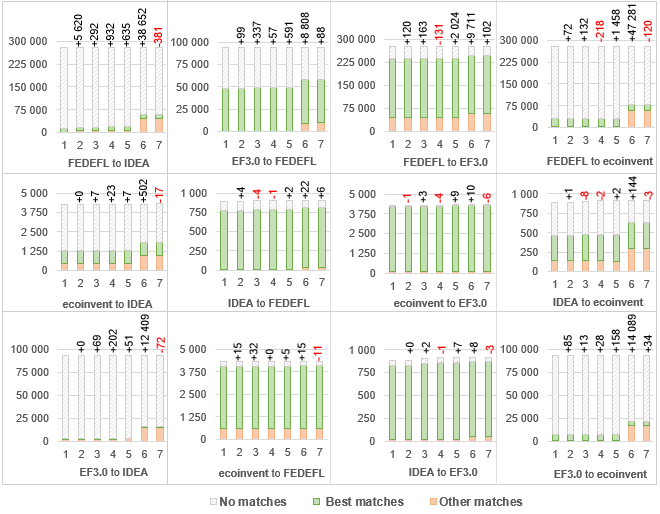


**Figure S3:** Coverage reached by each source-target list combination, broken down by type of match over the seven iterations. Positive figures over the bars represent the increase in total matches (i.e., regardless of whether “best” or “other” match type) with respect to the previous iteration. Negative figures (in red) represent a decrease in the total matches.
